# Etiology of Summer Diarrhea

**Published:** 1880-08

**Authors:** 


### ETIOLOGY OF SUMMER DIARRHEA.

AT a meeting of the Society of Medical Officers of Health, at 1 Adam street, Adelphi, London, on Friday, February 20th, president, Dr. Bristowe, Dr. G. B. Longstaffe read a paper entitled "Some facts bearing on the Etiology of Summer Diarrhea," of which the following is an abstract:—All are prepared to admit that many circumstances may predispose to diarrhea, such as the character of food, foul air, bad water, high temperature, overcrowding, etc.; but it is at least not improbable that there exists some special or specific cause, at present unknown, which must be superadded to one or more of the predisposing causes. This specific cause may be a meteorological condition, a peculiar form of pollution of air or water, some animal or vegetable matter in a state of change, or even a living germ. The method of inquiry employed by Dr. Longstaffe necessitates the limitation that when diarrhea is mentioned it must be understood that fatal diarrhea is meant, because the arguments to be brought forward are all derived from the statistics of death collected in the Registrar General's office. It is important to obtain satisfactory replies to the three following questions: Where, when, and how does it kill? The average death-rates of the ten years ending 1879 show that the average summer diarrhea death-rate over the whole of England and Wales is 2¼ per 1000; in the fifty other towns of the Registrar-General a little below 3 per 1000, while nearly 1 per 1000 higher than that of the twenty largest towns of England. The death-rate of all the rest of the country—that is, of the registration districts not included in these seventy towns—is 1½ per 1000. Diarrhea in the summer is, therefore, twice as fatal in the fifty towns, and more than twice as fatal in the twenty towns, as it is in the country districts. When the death-rate of the several towns is investigated, very striking differences come to light. Thus the death-rate in Leicester is over 8 per 1000, Preston 7 per 1000; next to Preston come Salford, Leeds, Hull, Yarmouth, Bolton, Liverpool, and Birmingham. The London rate is almost exactly the same as those of the fifty towns. When the seventy towns are arranged in the order of their summer diarrhea death-rate, the twenty towns at the top of the list are found to have an average rate of 5.2, while the twenty towns at the other end have an average of only 1.8. What factor in common have the twenty towns comprising one group, which being absent in the twenty towns comprising the other group, causes the latter to have a diarrhea death-rate of little more than one-third that of the former? Density of population is not the common factor; the geological system upon which a town stands appears to exert no influence, although porosity of soil may be of importance. Elevation above or proximity to the sea is without effect. Mortality from diarrhea is below the mean from the beginning of October to the end of June, while it very greatly exceeds the average during July, August and September. From December 1st to May 31st, the deaths from diarrhea in London are practically constant, averaging between fourteen and fifteen per week; three weeks after the mean temperature of the air rises above 50° F.—i. e., about June 1st—the deaths begin to get more numerous, and a fortnight after the temperature exceeds 60° F. the deaths begin to exceed the mean in London 52 per week. The greatest number of deaths occur in the first week in August, and the mortality appears to be due to some function of the temperature. Diarrhea is most fatal in years with hot summers, especially if they be at the same time dry, and is least fatal in cold summers, especially if they be at the same time wet. The number of rainy days appears to be at least as important as the amount of rain that falls. The temperature of the water of the

Thames has a closer relation to the mortality from diarrhea than the temperature of the air. The temperature of 62° F. is more critical than of 60° F. All attempts to express the diarrhea mortality as a function of temperature *only* have failed. Diarrhea is therefore probably a communicable zymotic disease, which thrives best during hot weather. From fifteen to twenty-five years of age the death-rate from diarrhea is only .04 per 1000 living at that age; from five to fifteen years of age it is a little greater, and from twenty-five to fifty-five only .1 per 1000. From two to five years it is .6 per 1000; over fifty-five years, 1.2 per 1000; during the second year of life 5.1 per 1000, and during the first year, 17 per 1000 living at that age. These figures are derived from the means of the years 1847–78. Dr. Longstaffe concludes that there is a specific poison of diarrhea which makes its appearance in the summer months, and that bad water, fruit, artificial feeding, etc., may be excluded as causes. There only remains then bad air, either (*a*) as a locally-bred miasm, or (*b*) sewer air. He suggests that the exciting cause of summer diarrhea is intimately connected with the process of putrefaction, however and wherever arising; but that in a large proportion of the cases the infective material has its source in the public sewers, and is introduced into the system through the lungs.—*London Lancet, June*, 1880.

